# Large emergent optoelectronic enhancement in molecularly cross-linked gold nanoparticle nanosheets

**DOI:** 10.1038/s42004-022-00723-2

**Published:** 2022-08-29

**Authors:** Steven Gravelsins, Myung Jin Park, Marek Niewczas, Seok-Ki Hyeong, Seoung-Ki Lee, Aftab Ahmed, Al-Amin Dhirani

**Affiliations:** 1 Department of Chemistry, University of Toronto, Toronto, ON Canada; 2 Department of Materials Science & Engineering, McMaster University, Hamilton, ON Canada; 3 Functional Composite Materials Research Center, Institute of Advanced Composite Materials, Korea Institute of Science and Technology (KIST), Wanju-gun, Jeonbuk-do Republic of Korea; 4 School of Materials Science and Engineering, Pusan National University, Busan, Republic of Korea; 5 Department of Electrical Engineering, California State University, Long Beach, CA USA

**Keywords:** Electronic properties and materials, Electron transfer, Chemical physics, Nanoparticles

## Abstract

A central goal in molecular electronics and optoelectronics is to translate tailorable molecular properties to larger materials and to the device level. Here, we present a method to fabricate molecularly cross-linked, self-assembled 2D nanoparticle sheets (X-NS). Our method extends a Langmuir approach of self-assembling gold nanoparticle (NP) arrays at an air-water interface by replacing the liquid sub-phase to an organic solvent to enable cross-linking with organic molecules, and then draining the sub-phase to deposit films. Remarkably, X-NS comprising conjugated oligophenylene dithiol cross-linkers (HS-(C_6_H_4_)_*n*_-SH, 1 ≤ *n* ≤ 3) exhibit increasing conductance with molecule length, ~6 orders of magnitude enhancement in UV-Vis extinction coefficients, and photoconductivity with molecule vs. NP contributions varying depending on the excitation wavelength. Finite difference time domain (FDTD) analyses and control measurements indicate that these effects can be modeled provided the local complex dielectric constant is strongly modified upon cross-linking. This suggests quantum hybridization at a molecule–band (q-MB) level. Given the vast number of molecules and nano-building blocks available, X-NS have potential to significantly increase the range of available 2D nanosheets and associated quantum properties.

## Abstract (web summary)

Bottom-up nanomaterial fabrication enables control over nano building-block structure and structure-property relationships, affording opportunities to engineer devices and materials with desirable electronic and optoelectronic behaviors. Here, the authors produce stable oligophenylene dithiol cross-linked gold nanoparticle nanosheets via interfacial self-assembly and show the emergent effects of the cross-linkers on the material’s electronic, optical and photoconducting properties.

## Introduction

Synthetic control over molecular structure and strong structure-property relationships in molecules afford an opportunity in molecular optoelectronics, namely a possibility to engineer molecular devices and materials with electronic/optoelectronic behavior that is tailored from the bottom-up^1,2^. Incorporating molecules into larger structures that continue to reflect control over single molecule properties, however, is an open challenge in this direction. Studies of molecular electronic devices have revealed that molecule–electrode contacts play a crucial role in influencing device behavior^1,2^, leading to significant device-to-device variation^1,3^. Further, Fermi-level pinning of molecular states by the electrodes can dominate electronic device behavior, repressing the effects of desirable individual molecular properties^1,2,4–6^. For instance, thiol-based conjugated molecules such as oligophenylene dithiols (OPDs) have exhibited decreasing conductance in molecular junctions with an increase in molecule length, although longer molecules within this family exhibit narrower highest occupied—lowest unoccupied molecular orbital (HOMO-LUMO) gaps^6^. The decrease in conductance with molecular length is attributed to Fermi-level pinning owing to gold–thiol bonds at the electrode–molecule interface, and is in contrast to the behavior exhibited by highly conjugated molecular wires such as fused porphyrins, where conductance across junctions increases with molecular length^7,8^. Encouragingly, however, reports of nanostructured materials comprising nanoparticles cross-linked with molecules have found that properties of such nanoparticle-molecule materials depend strongly on both nanoparticle and molecular building blocks used^9–11^. Moreover, nanoparticle-molecule cross-linked systems have shown that the incorporation of nanoparticles in molecular junctions can increase reproducibility of assembled devices^12^, as well as increase conductance across double layers of molecular self-assembled monolayers^13^. With a vast range of nanostructures and molecules available, cross-linked nanoparticle-molecule systems comprise a promising platform for the development of molecular optoelectronics. To date, however, a major bottleneck in this regard has been a lack of versatile methods for assembling ordered, extended cross-linked nanoparticle structures with a variety of cross-linking molecules. Nanoparticles are commonly first self-assembled either on a solid substrate^14,15^ or at an air–liquid interface^14,16,17^, before introduction of a molecular cross-linker to facilitate molecular exchange and linking between adjacent nanoparticles^9–11^. On solid substrates, nanoparticle cross-linking within the monolayer is generally restricted to molecules of lengths close to that of the interparticle separation^10,11^ in order to preserve the nanoparticle monolayer order/configuration^18^. Conversely, although nanoparticles assembled at air–liquid interfaces are highly mobile and interparticle distance can be controlled through Langmuir methods (e.g., varying surface pressure)^16^, cross-linking at such interfaces remains a challenge due to difficulties in introducing cross-linkers into the liquid subphase as well as facilitating ligand exchange. Since water is a common choice of subphase in such systems, hydrophobic cross-linkers such as organic dithiols and conjugated molecular wires cannot be introduced, and cross-linking is again restricted until the nanoparticle film is collected upon a solid substrate.

**Fig. 1:**
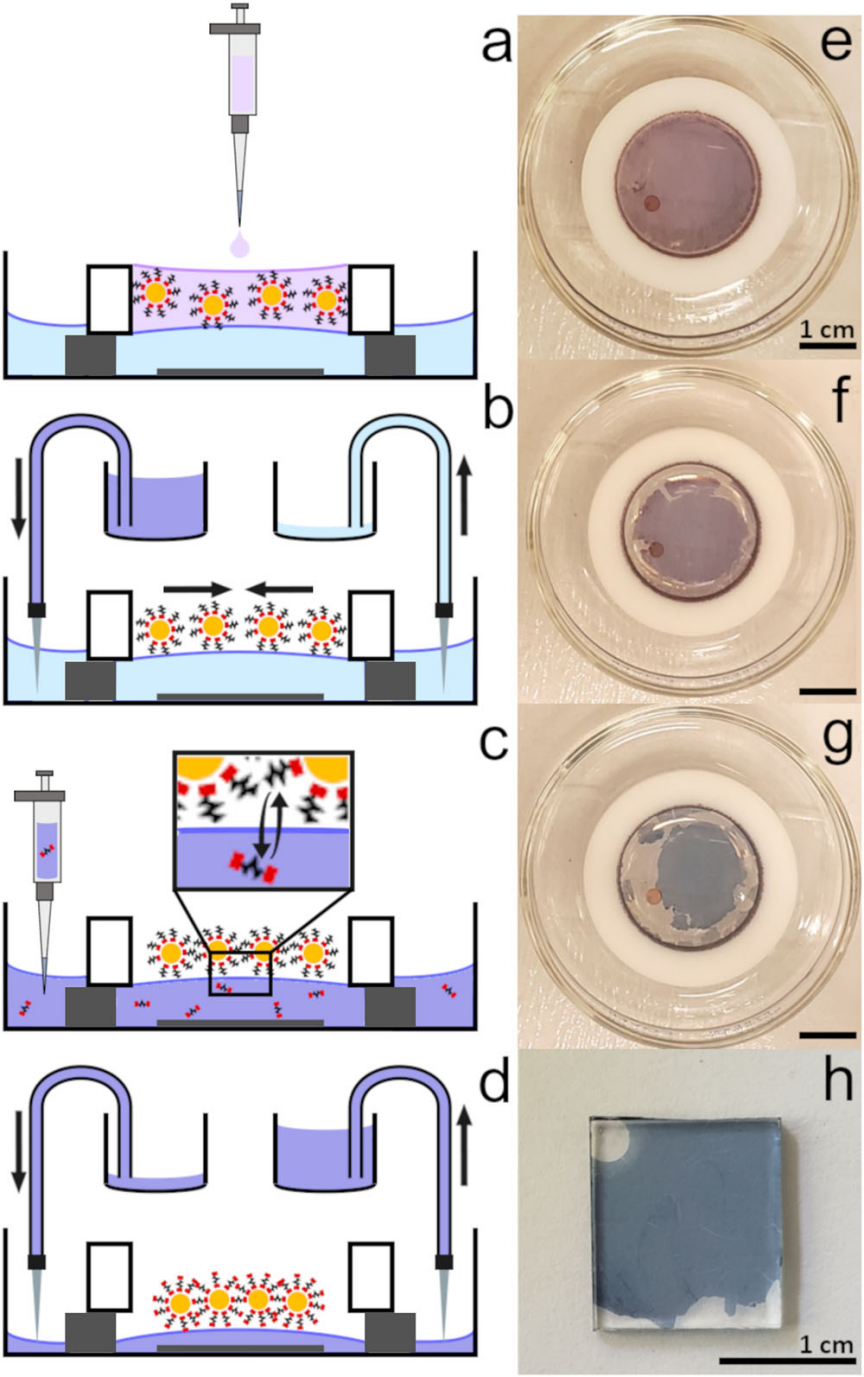
Illustration of the method used to fabricate X-NS. **a** Size-selected Au NPs are dispersed in hexane and drop cast on the water surface inside the Teflon ring (~2.51 cm inner diameter, supported by two 1 mm thick glass slides on each side, and positioned in a glass well with ∼6 cm diameter and ∼1.5 cm height). The Au NPs form a monolayer at the air–water interface as the hexane evaporates. **b** Methanol is pumped in and methanol/water is pumped out at ∼1.2 mL min^−1^ using two peristaltic pumps. **c** A molecular cross-linker is introduced into the methanol subphase. **d** The methanol molecular cross-linker solution is exchanged for neat methanol using two peristaltic pumps and finally the methanol subphase is drained to deposit the X-NS upon a substrate. **e**–**h** Photographs of the NP films formed in steps (**a**–**d**), respectively using 6.84 ± 0.61 nm Au NPs and butanedithiol cross-linker. All scale bars correspond to 1 cm.

The present work describes an extended Langmuir method that enables fabrication of molecularly cross-linked self-assembled nanoparticle sheets (X-NS) with a variety of aliphatic and conjugated organic dithiol cross-linkers of varying lengths. X-NS are fabricated by first self-assembling NP monolayers at an air–water interface and subsequently exchanging the water subphase for an organic solvent to enable in situ cross-linking with organic molecules *via* ligand exchange, as shown in Fig. 1. The subphase is then drained to deposit NP films upon a desired substrate. In particular, X-NS cross-linked with alkanedithiols (HS(CH_2_)_*n*_SH for *n* = 2, 4, 6, 8) and oligophenylene dithiols (OPDs) (HS-(C_6_H_4_)_*n*_-SH for *n* = 1, 2, 3), as well as densely packed gold nanoparticle monolayers ligated with oligophenylene thiols (H-(C_6_H_4_)_*n*_-SH for *n* = 1, 2) are investigated. This comparison approach enables insight into the effects of varying molecular backbones and varying the maximum number of strong gold–thiol interaction (on one side of the molecule *vs*. potentially on both). Structurally, these nanosheet materials are highly ordered, macroscopic in 2-dimensions and one nanoparticle thick. They exhibit electronic, optical and photoconducting properties that correlate with those of the gold nanoparticle and molecular cross-linker building blocks as well as reveal emergent effects. Remarkably, X-NS comprising conjugated OPD cross-linkers exhibit conductance that increases exponentially with molecule length due to electron delocalization, in contrast to previously reported OPD junctions using bulk electrodes^6^. Further, the rate of increase is 10-fold higher than that previously reported using similar molecules with terminal alkanethiol chains employed to isolate the conjugated molecular backbone from the substrate^19^. In addition, conjugated molecules in X-NS exhibit π → π^*^ transitions that red shift with increasing molecular length due to electron delocalization, as in solution, but with 6 orders of magnitude higher absorbance extinction coefficients. We use finite difference time domain analysis to model the absorbance of X-NS using the complex dielectric functions of the nanoparticle and molecular cross-linker building blocks as inputs and attribute emergent X-NS behavior to quantum hybridized molecule-band (q-MB) states.

## Results and discussion

**Fig. 2:**
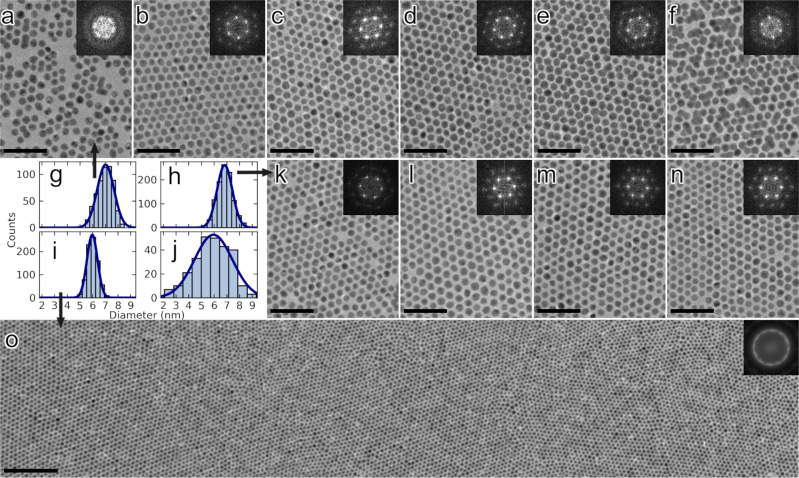
TEM characterizations of self-assembled NP films and X-NS. TEM images of NP films self-assembled without cross-linker deposited from (**a**) water and (**b**) methanol. **c**–**f** TEM images of X-NS cross-linked using *n*-alkanedithiols (*n*-HS(CH_2_)_*n*_SH with *n* = 8, 6, 4, 2, respectively. **g**–**i** Size distributions of fractionated NPs used to fabricate uncross-linked NP films or X-NS, as indicated with arrows. Average diameters are: 6.97 ± 0.60 nm (top row), 6.84 ± 0.61 nm (middle row), and 5.99 ± 0.41 nm (bottom row). **j** Size distribution of as synthesized NPs with an average diameter of 5.84 ± 1.39 nm. TEM images of (**k**) NP film self-assembled without cross-linker deposited from methanol and (**l**–**n**) X-NS with oligophenylene dithiol (OPD) cross-linkers (HS-(C_6_H_4_)_*n*_-SH with *n* = 3, 2, 1), respectively. **o** TEM of X-NS with benzene-dithiol cross-linker over multiple domains. Insets show 2-D Fast Fourier transforms of images in the respective main panels. Scale bars correspond to 40 nm in (**a**–**f**, **k**–**n**), and 100 nm in (**o**).

Figure 2 shows TEM images (main panels) with respective 2-D Fast Fourier transforms (FFT, insets) of various self-assembled NP films without cross-linker molecules and X-NS (Fig. 2a–f, k–o) as well as size distributions of NPs used (Fig. 2g–j). Figure 2a shows a TEM image of a film self-assembled on water. The film exhibits submonolayer coverage with gaps between NP domains. An FFT of the image (Fig. 2a, inset) exhibits 6 broadened peaks due to close-packed order within domains but some rotational variation between domains. Upon changing the liquid subphase to methanol, the gaps are much reduced, consistent with film shrinking observed on a macroscopic scale (Fig. 1e vs. 1f). Correspondingly, NP packing density and order increase significantly (Fig. 2b, k), and FFTs exhibit much sharper features as well as higher order peaks (Fig. 2b, k, insets). Further, switching to a methanol subphase also allows introduction of organic cross-linkers that are otherwise insoluble in water. Figure 2 shows TEM images of resulting X-NS and corresponding 2D FFTs obtained using n-alkanedithiols (Fig. 2c–f) and OPDs (Fig. 2l–n). Although inter-nanoparticle separation potentially decreases with *n*, nanoparticles generally remain isolated, except for X-NS obtained using the shortest alkanedithiols, namely butanedithiol and especially ethanedithiol (Fig. 2e, f). Such particle fusion is not observed in X-NS fabricated using aromatic dithiols (Fig. 2l–n). In all cases, 2-D FFT analysis reveals a high degree of local spatial order on a length scale of ~100 nm. Lower magnification TEM images reveal that the NP 2-D monolayer structure extends over at least micron length scales, and that the film is composed of several crystalline domains, randomly rotated with respect to one another (Fig. 2o). FFT analysis of these lower magnification areas produce a ring-like feature (Fig. 2o inset) due to averaging over the various crystalline hexagonally close-packed domains. These images show the monolayer architecture is preserved during the formation of the X-NS: 3D stacking/formation of aggregates is negligible.

**Fig. 3:**
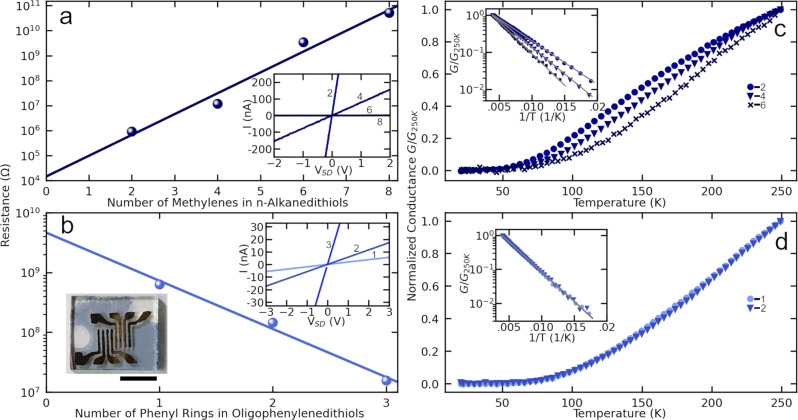
DC resistances of various X-NS. Average room temperature resistances of X-NS fabricated using (**a**) *n*-alkanedithiols and (**b**) oligophenylenedithiols. Standard deviations over several measurements are ~20% and are smaller than the size of the symbols. Insets show current-voltage curves for corresponding data in the main panels, and the photographic inset of (**b**) shows an X-NS fabricated using benzenedithiol and deposited on a glass slide with electrodes fabricated using thermal deposition. The scale bar corresponds to 5 mm. Conductance vs. temperature normalized to conductance at 250 K for various X-NS fabricated using (**c**) *n*-alkanedithiols and (**d**) oligophenylenedithiols. Numbers in insets of (**a**, **c**) indicate the number of methylenes in alkanedithiols while numbers in (**b**, **d**) indicate the number of phenyl rings in oligophenylene dithiols used to fabricate X-NS. Insets: Arrhenius plots of data in the corresponding main panels.

To explore the effect of dithiol cross-linkers introduced during X-NS fabrication, we measured X-NS electrical and optical properties, using films fabricated using monothiols as controls. Figure 3a, b contrast room temperature DC resistances of various X-NS fabricated using alkanedithiols and OPDs, respectively. The data are obtained by depositing X-NS onto gold electrode arrays thermally deposited onto glass substrates and measuring resistance between pairs of adjacent electrodes (electrode gaps = ~300 $\mu$m × ~3 mm, Fig. 3b inset photograph). Resistances increase exponentially as alkanedithiol chain length increases, consistent with an expected tunneling transport mechanism across the insulating alkanedithiol linker molecules^20,21^. The data are well described by an exponential of the form ${R}_{n}\,=\,{R}_{0}{e}^{\beta n}$ with *β* = 1.92 (*R*^2^ = 0.97), where *R*_*n*_ is the resistance of the n-alkanedithiol X-NS, *n* is the number of repeat units, and *R*_*0*_ and *β* are constants. This value of beta is larger than a typical value of ~1 per methylene unit reported by previous studies using one or a few molecules in break junctions or self-assembled monolayers on deposited electrodes^22–25^. In contrast, OPD cross-linked X-NS exhibit a reverse trend with DC resistances decreasing exponentially with increasing number of phenyl units. Again applying an exponential fit to resistance as above, this time data yields a negative value for *β* = −1.86 (*R*^2^ = 0.98) per phenylene unit (−0.44 ${\dot{A}}^{-1}$). Previous studies using highly conjugated molecules have reported widely varying resistance behavior due to sensitivity to electrode–molecule contacts^3^. For example, studies using self-assembled OPD monolayers on deposited electrodes have found increasing resistance with number of phenyl units which has been attributed to pinning of the Fermi-level closer to the highest occupied molecular level, which results in hole transport, tunneling and increasing resistance even as the gap between the frontier molecular energy levels decreases with increasing number of phenyl units^6^. Consistent with this picture, other studies have reported that oligothiophene π-systems can be decoupled from deposited electrodes by using a Van der Waals contact and/or inserting an alkyl spacer^19,26^. Although this is expected to increase molecule–electrode contact resistance significantly, an anticipated decrease in overall resistance with increasing number of thiophene units is observed. Values of *β* were estimated to be approximately ~−0.2 ${\dot{A}}^{-1}$, which is of the same order but smaller in magnitude than the value found in our system. With support from density functional theory, the negative *β’s* have been attributed to decreasing HOMO-LUMO gaps and gold Fermi level—molecular frontier orbital energy level barrier with increasing molecular length^19,26^. In the present study as well, decreasing HOMO-LUMO gap is observed as evidenced by red-shifting π–π^*^ absorption peaks (see molecule and X-NS UV–Vis spectra and discussion below). Decreasing gold Fermi level—molecular frontier orbital energy level barrier is also achieved in the present study without employing previously reported measures to decouple molecules from contacts^19,26^. Combined, these studies highlight critical roles that both the molecule and electrode–molecule architecture can play in governing the degree to which charge transport through molecular orbitals can be controlled by varying molecular structure, which is one of the central goals of molecular electronics. In particular, the present remarkable observation of a strong correlation between increasing delocalization at the molecular level and decreasing resistance at the X-NS level suggests that X-NS are a promising platform for the development of molecular electronic materials.

To explore whether the di-thiols generate a degree of NP cross-linking or simply replace capping ligands, we also measured the resistance of films fabricated using mono-thiols, namely benzenethiol and biphenylthiol, as controls (see Table 1 for summary and Supplementary Table 1 and Supplementary Fig. 9 for individual measurements). Films fabricated using benzene- and biphenyl-dithiols exhibit 2.3 and 7.3-fold lower resistance, respectively, compared with those fabricated using corresponding monothiols. Previous studies measuring electronic transport across junctions formed from oligophenylene thiols and dithiols also report greater conductance in dithiols vs. monothiols for molecules otherwise containing the same backbone, owing to metal-molecule chemical contacts on both sides of the junction^6,27^. These results suggest that cross-linking is indeed occurring in our system (i.e., at least some dithiols are binding from both sides to pairs of adjacent NPs, as opposed to all only binding to individual NPs), and film properties are significantly modified accordingly.

**Table 1:** DC resistance of nanoparticles films prepared using various molecules.

Terminal group	Thiol			Dithiol		
Molecular structure	Dodecane-	Benzene-	Biphenyl-	Benzene-	Biphenyl-	Terphenyl-
Average resistance (MΩ)	15,500 ± 25% (*N* = 3)	1500 ± 24% (*N* = 5)	1100 ± 19% (*N* = 5)	640 ± 26% (*N* = 5)	150 ± 24% (*N* = 5)	16 ± 15% (*N* = 5)

N is the number of samples.

Figure 3c, d show conductance vs. temperature data for alkanedithiol- and OPD-linked X-NS, respectively. In all cases, conductance increases with temperature and follows Arrhenius behavior (see insets for fits and Table 2 for activation energy barriers). Combined with the length dependence discussed above, the overall conductance is ${G}_{n}\,=\,{G}_{0}{e}^{-\beta n}{e}^{-{E}_{a}/{k}_{B}T}$, where ${k}_{B}$ is Boltzmann’s constant and $T$ is absolute temperature. For alkanedithiols, the activation barriers are consistent with single electron charging behavior^28^. Using a simple 3-D model, the energy (${E}_{a}$) required to add a single electron to a NP surrounded by a dielectric layer representing molecular linkers and a metal shell representing the remaining film is ${E}_{a}\,=\,{e}^{2}s/8\pi {\varepsilon }_{0}\varepsilon r(r\,+\,s)$, where *r* is the radius of the NP, *s* is the thickness and *ε* the dielectric constant of the dielectric layer and $r\,+\,s$ is the radius of the metallic shell. This simple model provides useful insight but underestimates the charging energy since the surrounding NPs in X-NS form a 2-D film rather than a 3-D shell as assumed; as a result, here the electric field above and below actually extend beyond a distance, $s$, assumed by the model. Nevertheless, using typical values $s$ = 0.5 nm, $r$ = 3.2 nm and $\varepsilon$ = 2 yields ${E}_{a}$ = 15 meV, which is of the correct order. Also, the model predicts that ${E}_{a}$ should increase with $s$ as observed. In contrast, ${E}_{a}$ for OPD-linked X-NS do not change with increasing length. This is further confirmed by noting that conductance vs temperature data for phenyl- and biphenyl-dithiol linked X-NS overlap each other when normalized to respective conductance at room temperature. The observed barrier may arise from defects such as domain boundaries; however, further study is required in this direction.

**Table 2:** Activation energies extracted from Arrhenius plots shown in Fig. 3c, d.

Molecular structure	Ethanedithiol	Butanedithiol	Hexanedithiol	Benzenedithiol	Biphenyldithiol
Activation energy (meV)	23	29	34	35	35

**Fig. 4:**
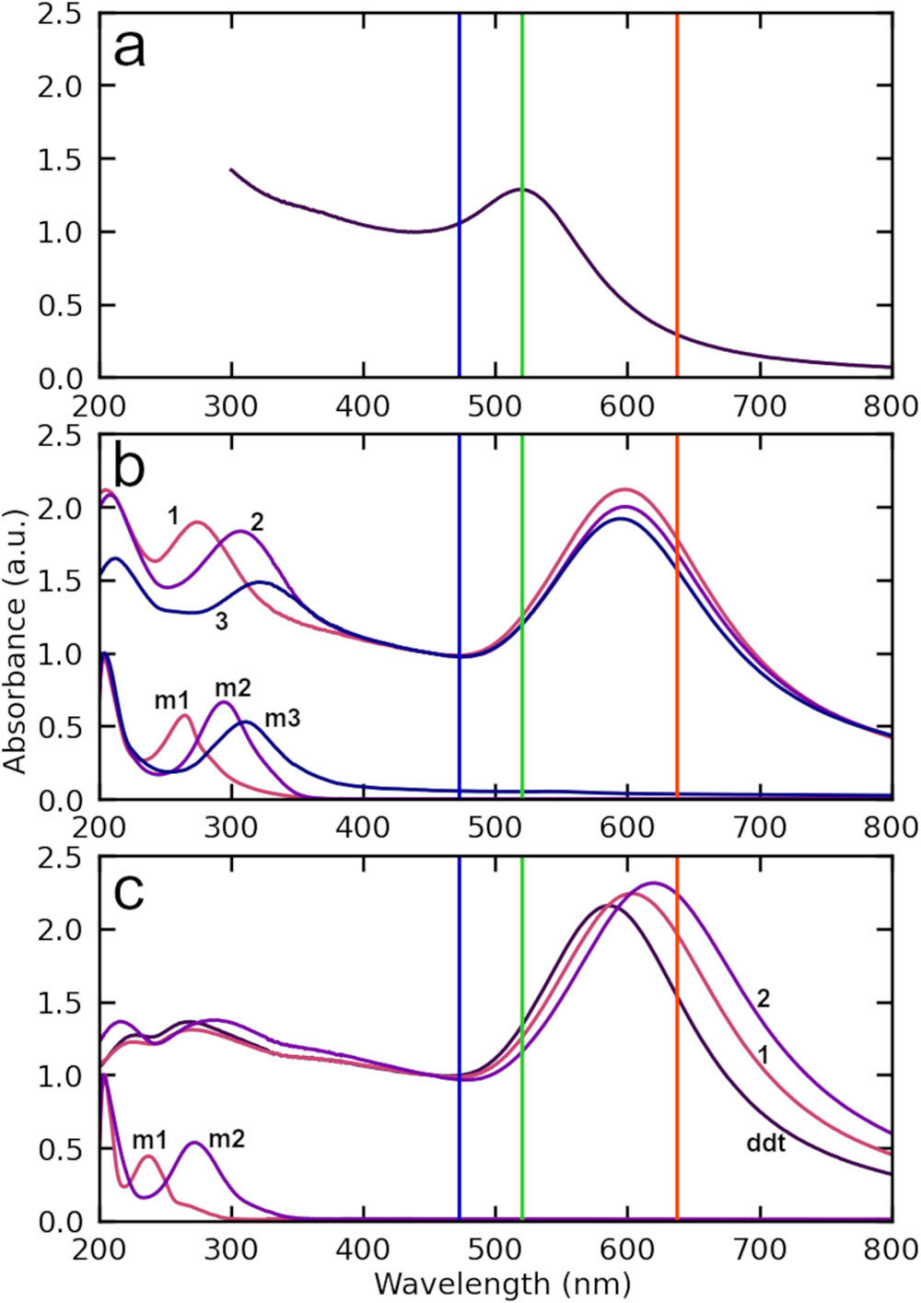
Normalized UV–Vis absorbance spectra of various molecules in solution, NP films and X-NS on quartz prepared using various molecules. **a** NPs in a hexane solution. **b** X-NS prepared using oligophenylenedithiols (HS-(C_6_H_4_)_*n*_-SH with *n* = 1, 2, 3) and corresponding oligophenylenedithiols in solution (m1, m2, and m3, respectively). **c** NP films prepared using dodecanethiol (ddt), phenyl- and biphenyl-thiol (H-(C_6_H_4_)_*n*_-SH with *n* = 1, 2) and corresponding conjugated thiols in solution (m1 and m2, respectively). Blue, green and red lines denote 473, 520, and 638 nm excitation wavelengths, respectively, used for photoconductivity measurements discussed below. See Supplementary Information for UV–Vis spectra prior to normalization.

Ultraviolet–visible spectroscopy (UV–Vis) provides further evidence for phenomena enabled by cross-linking in this system. Figure 4 shows that varying molecular backbone structure (i.e., alkane vs. oligophenylene and molecule length) as well as molecular terminal functionality (mono- vs. di-thiols) both have strong effects on the absorbance of NP films. There are two distinct spectral regions of interest. We first consider the longer wavelength absorption peaks (~570 to ~640 nm) attributed to plasmonic response of the metal nanoparticle arrays. The spectral location of this peak is highly sensitive to the size of the gap between adjacent nanoparticles, the complex dielectric constant of the inter-nanoparticle medium, and the sizes of the individual nanoparticles^29^. OPD X-NS exhibit NP surface plasmon peaks that are all significantly red-shifted compared to films with dodecanethiol capped NPs (~600 nm vs. 570 nm, respectively), but exhibit little further shifting within the series as the number of phenyl units is varied (Fig. 4b). In contrast, oligophenylenethiol NP films do exhibit surface plasmon red shifts with increasing number of (phenyl) units (Fig. 4c) compared with dodecanethiol NP films: from 600 to 620 nm for benzene- and biphenyl-thiols. Comparing the effect of molecular backbone chemical structure, alkanedithiol X-NS exhibit NP surface plasmon peaks that gradually red shift from 580 to 640 nm as the number of methylene units decreases from 8 to 2 (see Supplementary Figs. 7 and  8 for UV–Vis spectra of another set of X-NS fabricated with OPDs as well as alkanedithiols). This trend is consistent with reported plasmon red-shifting as interparticle distance is reduced in NP clusters or arrays^18,29,30^. That both the conjugated X-NS and NP films do not follow expected red-shifting of plasmon peaks with decreasing molecule length is indicative of additional phenomena and a significant change in the optical properties of the inter-nanoparticle medium—discussed further below.

The shorter wavelength absorptions peaks of OPD X-NS in Fig. 4b are also of particular interest. The spectral location of these peaks coincides with molecular absorption spectra: peaks at 280–320 nm can be attributed to π–π^*^ transitions (the shift to longer wavelengths reflects increasing electron delocalization) and the peak at 200–210 nm to the sulfur moiety absorption. Remarkably, the strengths of these molecular absorption peaks are prominent on the scale of the NP surface plasmon peaks even though the NPs are much larger, are metallic and respond collectively. For reference, molecular absorption peaks in NP films with corresponding oligophenylene monothiols are much weaker (Fig. 4c). Given that the UV–Vis absorbance of oligophenylene monothiols and dithiols in solution are similar (see Supplementary Fig. 6 for in solution-phase spectra), we attribute this unexpectedly large molecular absorption in X-NS and resistance data discussed above to emergent phenomena enabled by cross-linking. These remarkable features are explained with the help of a material model discussed in detail below.

To study OPD cross-linked X-NS absorption in more detail, we use finite difference time domain (FDTD) analysis and compare absorption of systems with monothiol ligands vs. dithiol cross-linkers and phenyl- vs. biphenyl- molecular backbones. To highlight emergent effects, we rely on measured optical properties of building blocks, i.e., measured UV–Vis absorption spectra of molecules in solution and published complex dielectric constant of bulk gold, as inputs, and model corresponding properties of molecule-NP assemblies. Although this approach is approximate, for instance it neglects solvent contributions and variation in molecule response within the inter-NP region, the simplicity and effectiveness of the model provide useful insight into behavior exhibited by X-NS. Rearranging the Beer-Lambert law for a lossy medium, molecule absorbance is given by:

1 $$A\,=\,\left({{{{{{\rm{log }}}}}}}_{10}e\right)\frac{4\pi {\kappa }_{m}l}{\lambda }\,=\,\,\varepsilon {cl}$$

where $l$ is the path length of the light in the medium, $c$ is the concentration of the molecule, $\varepsilon$ is the molar absorptivity constant, $\lambda$ is the wavelength of light and ${\kappa }_{m}$ is the molecular extinction coefficient (imaginary component of the molecular complex index of refraction, ${N}_{m}$). This formula is used to calculate the molecular extinction coefficients from absorbance spectra measured with molecules in solution, and Kramers-Kronig (K-K) relations are then employed to approximate the corresponding real part of ${N}_{m},{n}_{m}$:

2 $${n}_{m}\left(\omega \right)\,=\,\,\frac{1}{\pi }P\int d\widetilde{\omega }\frac{{\kappa }_{m}(\widetilde{\omega })}{\widetilde{\omega }\,-\,\omega }$$

3 $${N}_{m}\left(\omega \right)\,=\,{n}_{m}\left(\omega \right)\,+\,i{\kappa }_{m}\left(\omega \right)$$

**Fig. 5:**
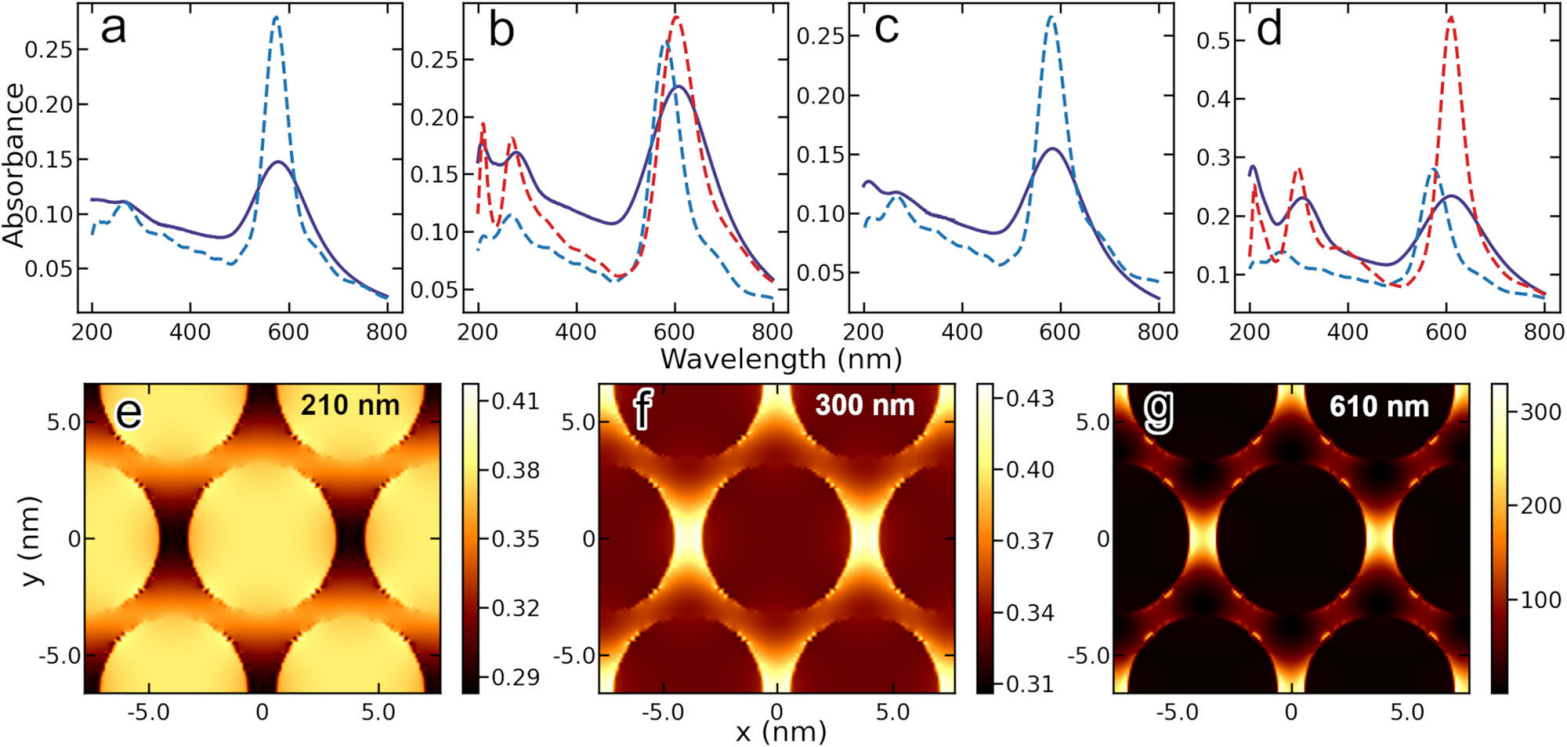
UV–Vis spectra and finite difference time domain modeling for nanoparticle films fabricated using various molecules. Solid lines: experimental UV–Vis absorption spectra of films assembled with 5.99 ± 0.48 nm NPs. Dashed lines: numerical model including the effects of both conductivity, σ, and molecular extinction factor, ${\kappa }_{m}$, with enhancement factor, *Λ*. **a** benzenethiol (blue: *σ*  = 4 × 10^3^ and *Λ* = 1), (**b**) benzenedithiol (blue: *σ* = 6 × 10^3^ and *Λ* = 1; red: *σ* = 10 × 10^3^ and *Λ* = 1.4 × 10^6^), (**c**) biphenylthiol (blue: *σ* = 6 × 10^3^ and *Λ* = 1), and (**d**) biphenyldithiol (blue: *σ* = 6 × 10^3^ and *Λ* =  1; red: *σ* = 12 × 10^3^ and *Λ* = 1.7 × 10^6^). Modeled normalized electric field intensity (|**E**|/|**E**_0_|)^2^ maps for biphenyldithiol cross-linked films at (**e**) 210 nm, (**f**) 300 nm and (**g**) 610 nm. Incident electric field is *x*-polarized.

It is noted here that the K-K relations provide approximate values of ${n}_{m}$ due to the limited spectral range of ${\kappa }_{m}$. The approximation can be considered valid over most of the spectral range of interest except near the edges. To account for conduction through the molecules, the complex dielectric constant of the molecule (${\epsilon }_{m}\,=\,{N}_{m}^{2}$) is modified by adding a conductivity term, $\sigma :{\epsilon }_{m}^{{\prime} }\,=\,{\epsilon }_{m}\,+\,i\frac{4\pi \sigma }{\omega }$. Here $\sigma$ is the conductivity which is adjusted to better fit the experimental results and to provide insight into the physical phenomena responsible for the optical spectra observed. Gold NPs are modeled using the complex refractive index of gold ${N}_{{Au}}$ obtained from the published values for bulk gold^31^. Comprehensive 3D-FDTD simulations are conducted to solve Maxwell’s equations and determine overall film absorption and local fields. We first calculate the absorption spectra of NP films fabricated using mono-thiol molecules, which represents a simpler case where the short wavelength molecular peaks are much less pronounced. Numerically calculated absorption spectra for benzene- and biphenyl-mono-thiol NP films are shown in Fig. 5a, c respectively (blue dashed lines) along with the experimental results (solid lines). Theoretical and experimental results are in relatively good agreement except the experimental absorption peaks are broader and slightly weaker in amplitude, which is attributed to NP size dispersion and gap variations (see Supplementary Fig. 10 for simulations varying NP size and interparticle spacing). Another deviation is seen near the edges of the spectral range which is a result of band-limited K-K relations. The absorption peak of the biphenyl-thiol film (Fig. 5c) is slightly red-shifted compared to that of benzene-thiol (Fig. 5a). The observed red shift is counterintuitive as one would expect a larger interparticle gap in the biphenyl-thiol film and thus resulting in a blue shift. Our numerical results explain the red shift as being due to increased refractive index of the biphenyl-thiol film caused by increased conductivity.

A remarkable feature of benzene- and biphenyl-dithiol X-NS absorption spectra are the large molecular absorption peaks that are on the scale of NP surface plasmon resonance peaks. The above approach of introducing molecular conductivity fails to model the observed spectra for these di-thiol X-NS (blue dashed lines vs. solid lines in Fig. 5b, d, respectively): attempting to improve the model at shorter wavelengths using significantly larger values of conductivity leads to much larger values of ${\kappa }_{m}$ and *n* causing the main plasmon absorption peak to broaden and red shift significantly, thus making it impossible to model both short and long wavelength absorption peaks simultaneously. However, if, in addition to increasing conductivity, ${\kappa }_{m}$ is increased by a large multiplication factor, Λ (~10^6^), then we obtain much better agreement between the model and observed spectra for both benzene- and biphenyl-dithiol X-NS (red dashed lines vs. solid lines in Fig. 5b, d, respectively). We note that such a large ~10^6^-fold absorbance enhancement factor is not observed with the corresponding mono-thiol NP films, which serve as a control, and cannot be attributed to local field enhancements, which the model accommodates. Also, the absorption enhancement cannot be attributed to differences in number of molecules in solution vs. NP film probed by the UV–Vis spectrometer: we estimate that ${cl},$ the number of molecules per unit area (nm^−2^) perpendicular to the beam path is 60 in solution, 10 in X-NS and 0.3 in hot-spots in X-NS (that is, (size of the hot-spot)^2^/(size of the nanoparticle)^2^ ≈ (1 nm)^2^/(6 nm)^2^ fewer than total in X-NS, see Fig. 5f discussed below and estimates in Supplementary Notes). The large increase in ${\kappa }_{m}$ is necessary according to Eq. 1 since conjugated dithiol absorbance in X-NS remains strong even though ${l}$ decreases from 1 cm in solution to ~6 nm (X-NS thickness) or ~1 nm (hot-spot thickness). ${\kappa }_{m}$ is proportional to $\varepsilon c$, and we estimate that both terms contribute to the increase in ${\kappa }_{m}$ as follows. Using the above values for number of molecules/area (${cl}$) and dividing by corresponding $l$’s, shows that concentration increases by 5 × 10^4^–3 × 10^5^ going from solution to X-NS. Using biphenyldithiol as an example, the absorbance at 300 nm decreases from 0.17 in solution (Supplementary Fig. 6) to 0.06 in X-NS (Supplementary Fig. 6). Dividing these absorbances by ${cl}$ yields a 2 to 65-fold increase in $\varepsilon$ depending on whether all molecules in X-NS or just those in hot-spots contribute significantly to the absorbance. The actual increase in $\varepsilon$ is likely in between or about ~10-fold. This is consistent with an observed ~10-fold higher absorbance at 200 nm for biphenyl-thiol (Fig. 5c) vs. -dithiol (Fig. 5d). The estimated ~5 orders increase in *c* and ~1 order increase in $\varepsilon$ combine to give ~6 orders increase in ${\kappa }_{m}$ consistent with the increase in ${\kappa }_{m}$ required by fitting.

To gain further insight into the nature of enhanced molecular absorbance exhibited by X-NS, ${\kappa }_{m}$ can be written as:

4 $${\kappa }_{m}^{2}\,=\,\,\frac{{\mu }_{m}}{2}\left({\left[{{\epsilon }_{m}}^{2}\,+\,{\left(\frac{4\pi \sigma }{\omega }\right)}^{2}\right]}^{1/2}\,-\,{\epsilon }_{m}\right)$$

and in a Lorentz oscillator model ${\epsilon }_{m}$ is given as:

5 $$\frac{{{\epsilon }}_{m}}{{\epsilon }_{0}}\,=\,1\,+\,{\omega }_{p}^{2}\mathop{\sum}\limits_{i}\frac{{f}_{i}}{{\omega }_{0,i}^{2}\,-\,{\omega }^{2}\,-\,i\omega {\gamma }_{i}}$$

where ${\mu }_{m}$ is the magnetic permeability, ${\omega }_{p}\,=\,\sqrt{\frac{N{e}^{2}}{{\epsilon }_{0}m}}$ is the plasma frequency, $N$ is the number of electrons/volume, $m$ is mass, ${\omega }_{0,i}$ is the resonance frequency, ${\gamma }_{i}$ the damping coefficient and ${f}_{i}$ the oscillator strength of the i’th transition. ${{\epsilon }}_{m}$ is a measure of the molecular polarization (dipole moment/volume) induced by an electric field. This suggests that upon cross-linking, both conductivity and ${\epsilon }_{m}$ increase for conjugated dithiols, possibly due to charge transfer with metallic NPs as near resonance, the model predicts ${\kappa }_{m}\,\propto\, \sqrt{N}$. As such, the resulting large and red-shifted molecular absorption resonance has both metallic and molecular characters, likely mediated by q-MB states (further support is provided by photoconductivity vs. excitation wavelength data discussed below).

Figure 5e–g show electric field intensity maps at resonances (210, 300, and 610 nm, respectively) for biphenyldithiol X-NS excited by an x-polarized incident field. Bright and dark regions represent strong and weak field intensities, respectively. These results illustrate collective modes of X-NS and corresponding UV–Vis absorption features. The model predicts that field intensities are strongest within the NPs at 210 nm (even though it is a resonance associated with the sulfur moiety on the NP surface) and between NPs at 300 nm (π–π^*^ transition). At 610 nm (~surface plasma resonance), field enhancement is concentrated in regions where NPs are closest, also known as hot-spots. These results combined with TEM structural data indicate that the optoelectronic response of X-NS is dominated by dithiols located in the regions of closest NP-NP approach, where dithiol—NP cross-linking is most likely.

**Fig. 6:**
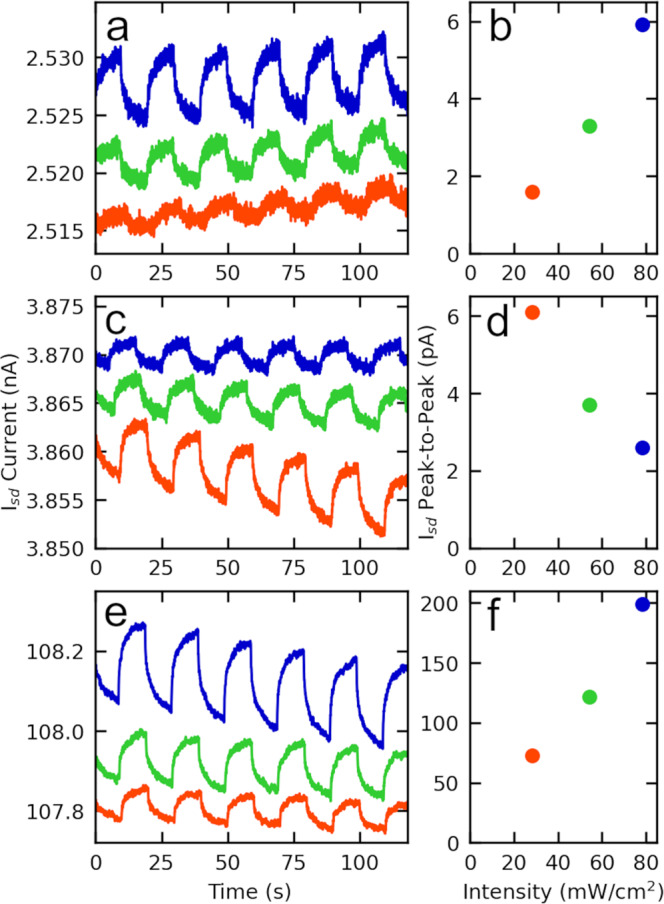
Photoconductivity vs. time as the excitation laser is repeatedly switched on/off and peak-to-peak photocurrent current vs. laser excitation intensity, respectively, for various NP films and X-NS. **a**, **b** benzenethiol NP film, (**c**, **d**) benzenedithiol X-NS, and (**e**, **f**) terphenyldithiol X-NS. Excitation laser power/area and wavelengths are 78.2 mW cm^−2^ at 473 nm (blue data/top curves in (**a**, **c**, **e**) offset by ∼+0.2% for clarity), 54.3 mW cm^−2^ at 520 nm (green data/middle curves in (**a**, **c**, **e**) and 28.2 mW cm^−2^ at 638 nm (red data/bottom curves in (**a**, **c**, **e**) offset by ~–0.2% for clarity). The spot diameter in all cases is ~3 mm. Current is measured using 2 probes and 2 V applied bias.

Figure 6 shows that NP films prepared using various aryl-thiols and -dithiols are photoconducting. Three excitation wavelengths are used in these measurements: 473 nm (78.2 mW cm^−2^), 520 nm (54.3 mW cm^–2^) and 638 nm (28.2 mW cm^−2^) (the wavelengths are shown as blue, green, and red lines, respectively, in UV–Vis spectra given in Fig. 4). Comparing the effect of thiol vs. dithiol, benzenethiol NP films exhibit largest photoconductivity responses at 473 nm (highest power) and the smallest at 638 nm (lowest power), respectively. Corresponding photon energies are too low to excite transitions between frontier molecular orbitals, and photoconductivity correlates with excitation laser power (Fig. 6b). Accordingly, we attribute photoconductivity to energy absorption by Au and thermally activated charge transport over small barriers. In contrast, benzenedithiol X-NS exhibit the opposite trend with 638 nm (lowest power) generating the largest photoconductivity. The 638 nm excitation wavelength is close to the surface plasmon resonance of the benzenedithiol X-NS, and modeling discussed above shows that fields between NPs and at the NP surface are strong. Accordingly, we attribute the photoconductivity also to thermally assisted transport across barriers but in this case mediated by surface plasmon resonance absorption^32–34^. Note that photoconductivity data (background current at 2 V in Fig. 6) and DC resistance data (Table 1) indicate that benzenedithiol films are 1.5- to 2.5-fold more conducting than benzenethiol films, respectively. At the same time, the UV–Vis enhancement factor, Λ, for benzenedithiol X-NS is 6 orders higher than for benzenethiol, and modeling discussed above indicates that this plays a key role in red shifting the plasmon resonance frequency significantly to 610 nm, close to the 638 nm excitation wavelength (Fig. 5). Varying molecular backbone within the dithiol series, going from benzenedithiol-linked to terphenyldithiol-X-NS, the photoconductivity response trend reverses, again following the expected correlation with power. Note also that current at the same applied bias increases ~30-fold (Fig. 6b, c). This is of the same order as a 40-fold reduction in DC resistance given in Table 1. This increase in conductance likely plays an important role in restoring the correlation between laser power and photoconductivity i.e., charges delocalized through conjugated molecular orbitals contribute to photoconductivity of X-NS, even though photon energies are too low to generate transitions between frontier molecular orbitals. This underscores that quantum hybridization between molecule and NP states is expected to be critical in X-NS. As photon energy continues to increase, eventually molecular resonances can be excited, carrier densities can increase, and photoconductivity is expected correspondingly to increase further^11^. For reference we note that NP films prepared using dodecanethiol exhibited no measurable photoconductivity response at any of these wavelengths (see Supplementary Figs. 11–17 for photocurrent measurements of other NP films and X-NS samples). Such variation of X-NS photoconductivity behavior with molecular structure, combined with underlying emergent transport and optical data presented above, show that these hybrid organic molecule—metal NP sheets are a promising platform for molecular optoelectronics.

## Conclusion

In summary, we have demonstrated a bottom-up method to fabricate extended molecularly cross-linked 2-D nanoparticle sheets (X-NS) with a variety of aliphatic and conjugated dithiol cross-linkers of varying lengths. In particular, oligophenylene monothiols and dithiols (OPDs) as well as alkanedithiols are incorporated into this system and resulting electronic and optical properties of the films are characterized. Room temperature resistance measurements reveal exponentially decreasing conductance with increasing alkanedithiol lengths in X-NS, and conversely exponentially increasing conductance with increasing oligophenylene dithiol length. Additionally, UV–Vis measurements of OPD X-NS exhibit increased molecular absorbance in comparison to films fabricated with oligophenylene monothiols and molecules in solution. FDTD modeling of OPD X-NS suggest that the enhanced absorption in such films can be explained by a ~10^6^-fold increase in molecular extinction coefficients, pointing to a strongly modified local complex dielectric constant upon cross-linking, which we argue is due to quantum hybridization at a molecule—nanostructure level. With a vast range of molecules and nano-building blocks available, as well as the versatility of the presented extended Langmuir method, X-NS have a potential to increase the range of available 2-D nanosheets and associated quantum properties.

## Methods

### Gold nanoparticle synthesis

Au NPs were synthesized according to a modified Brust method^28,35,36^. 50 mL of 50 mM tetraoctylammonium bromide in toluene was added to 25 mL of 40 mM hydrogen tetrachloroaurate in water and stirred for roughly 20 min. The colored organic phase was then removed, and added to 25 mL of 0.4 g of sodium borohydride dissolved in water, and stirred under vigorous agitation for 18 h. The deep red organic phase was then separated again, and washed 3 times with 50 mL each of aqueous 0.1 M H_2_SO_4_, 1 MNa_2_CO_3_, and finally deionized water. Anhydrous magnesium sulfate was then used to remove excess water from the organic nanoparticle solution. The resulting stock Au NPs possessed diameters of ~ 5.8 nm ± 24% and tetraoctyl-ammonium bromide ligands in toluene. UV–Vis spectra of the stock Au NPs is shown in Supplementary Fig. 1 (for TEM and histograms of particle sizes, see Fig. 2).

### Size-selection of gold nanoparticles

As synthesized Au NPs were subsequently size-selected^36^ before use in X-NS fabrication. 0.042 g of dodecylamine (DDA) was added to 5 mL of stock NP solution, and left to sit for at least 30 min for ligand exchange to occur. Simultaneously, a dodecylamine/ethanol (nonsolvent) solution was prepared by adding 0.214 g of DDA to 10 mL of absolute ethanol. The nonsolvent solution was then slowly added to the NP solution while simultaneously stirring the mixture. Once a volume ratio of 5 (mL): ~3.5 (mL) toluene: DDA/EtOH was achieved, the NP solution was centrifuged for 10 min at 2400 rpm (900 g). The precipitate was then collected, while more ethanol nonsolvent was added to the supernatant for further size-selection of the nanoparticles, collecting precipitates at volume ratios of 5 (mL): ~7 (mL) and 5 (mL): ~10 (mL) of toluene: ethanol. Note that the amount of ethanol nonsolvent added prior to each centrifugation step can be adjusted to tune NP precipitate sizes and yields. UV–Vis spectra of size-selected NPs are shown in Supplementary Fig. 2. NPs obtained at a volume ratio of 5 (mL): ~3.5 (mL) toluene : DDA/EtOH were used to fabricate the X-NS cross-linked with alkane-dithiols characterized in Fig. 2. Finally, the dodecylamine ligands encapsulating the size-selected NPs were exchanged for dodecanethiols. Size-selected NP precipitates were dispersed in hexanes, and injected with 0.25 mL of a solution of 0.25 mL of dodecanethiol in 5 mL of hexanes. Redispersed NP precipitates were left for ~1 h for ligand exchange to occur. Subsequently, size-selected NP solutions were evaporated under vacuum, washed several times with methanol, and stored as precipitates until their use in fabricating NP films or X-NS.

### X-NS and NP film fabrication

#### Formation of Au NP monolayers

Figure 1 illustrates the method used to fabricate X-NS. The first step in nanosheet fabrication is the generation of an ordered Au NP monolayer using a Teflon ring apparatus^17,36^. A Teflon ring (~2.51 cm inner diameter, supported by two 1 mm thick glass slides on each side) is positioned within a glass well (~6 cm diameter and ~1.5 cm height) and slowly filled with deionized water until the water surface exhibits a slightly convex surface curvature (see Fig. 1a and Supplementary Fig. 3). Both the Teflon ring and the glass well in which it is placed are housed in a transparent plexiglass box to minimize disruptions from air currents during X-NS fabrication (see Supplementary Fig. 4). ~6 nm dodecanethiol-ligated size-selected Au NPs are prepared for X-NS fabrication by diluting a size-selected precipitate (described above) in hexane such that the solution exhibits a UV–Vis absorbance of ~0.1 at 400 nm through a 1 cm path length. 0.9 mL of such a NP solution is then drop cast on the water surface inside the Teflon ring. As the hexane evaporates, the convex curvature of the water surface causes a meniscus between the hexane and water to form and grow radially outwards from the center of the Teflon ring to its wall. Simultaneously, the NP concentration increases, and the NPs deposit radially outward, affording improved NP packing. An ordered, close-packed NP monolayer self-assembles over centimeter scales as the hexane evaporates over about 30 min (see Fig. 1a).

#### Subphase exchange

Although this Langmuir approach enables NPs with hydrophobic organic (e.g., alkanethiol) capping groups to move on the water surface and form a relatively dense and ordered monolayer (Fig. 1e), cross-linking or, more generally, substitution with molecules is restricted as only molecules that are soluble in the aqueous subphase can be used. In addition, organic capping groups, residual hexane and/or excess alkanethiol ligands between NPs should be removed from the monolayer to encourage cross-linking. As such, an aqueous subphase is problematic as it prevents removal of such water insoluble molecules from the NP monolayer. Thus, to enable cross-linking of the NPs, we add a step of employing peristaltic pumps to slowly exchange the water subphase supporting the self-assembled NP monolayer with a polar organic solvent (see Fig. 1b). Methanol is chosen here because it is both miscible with water and is a reasonably good solvent for many organic molecules. Following the formation of a NP monolayer at the air–water interface, methanol is flowed into the subphase on one side of the glass well at a rate of ~1.2 mL/min for 30 min, while the water/methanol mixture is simultaneously removed from the other side of the well at the same rate (see Supplementary Fig. 4). The flow rates are carefully adjusted to ensure the subphase height remains approximately constant during the exchange. The self-assembled NP film visibly shrinks in area during this step presumably because, as mentioned above, organic molecules which are soluble in methanol but not water are removed (Fig. 1f).

#### Cross-linking

At this stage molecular cross-linkers are introduced into the methanol subphase (Fig. 1c). For cross-linkers (e.g., terphenyl-dithiol) that are less readily soluble in methanol, the methanol subphase is replaced with a dilute solution of the dithiol cross-linker in methanol using the same pumping method/apparatus (~0.1–0.5 mM, flow rate ~1.2 mL/min for ~40 min). Alternatively, for cross-linkers that are more readily soluble in methanol (e.g., butane-dithiol), the initial methanol exchange is performed for an hour instead of 30 min, after which a concentrated cross-linker solution is injected using a micropipette so as to produce a ~0.5 mM final concentration in the ~10 mL methanol subphase. After introduction of the cross-linker, the Teflon ring/glass well apparatus is covered with a large petri dish to slow methanol evaporation as the NP films are left for several hours or overnight to allow ligand exchange and NP cross-linking. Figure 1f, g show that this step causes the NP film to shrink further and visibly change color, appearing distinctly bluer.

#### X-NS collection

Following NP cross-linking, the methanol/cross-linker subphase is once again exchanged by flowing neat methanol (~30 min at ~1.2 mL/min) to remove excess dithiols, and the subphase is then drained (~1 mL/min), depositing the film on a desired substrate located at the bottom of the well (Fig. 1d, Supplementary Fig. 5). Following deposition, NP films are left in the plexiglass box undisturbed for ~1 h to allow remaining methanol to evaporate. This drain-to-deposit approach allows X-NS to be deposited on a desired substrate without shattering the X-NS. We note that X-NS stored under argon and at room temperature remain stable over at least a month-long timescale, and X-NS fabricated with longer molecular cross-linkers (hexane-dithiol, octane-dithiol, and all OPDs studied here) remain stable for at least several months. After these timescales, adjacent NPs may begin to fuse/the degree of fusion may increase, with this effect being more pronounced in NP films with shorter molecules and monothiols.

## Supplementary information

Supplementary Information

## Data Availability

All data generated or analysed during this study are included in this published article and its  Supplementary Information files.
