# Supplementary material for: Large emergent optoelectronic enhancement in molecularly cross-linked gold nanoparticle nanosheets
Source: Commun Chem. 2022 Aug 29;5:103. doi: 10.1038/s42004-022-00723-2 (PMC9814044; doi:10.1038/s42004-022-00723-2)
Supplement: Supplementary file 1 — Supplementary Information [file 42004_2022_723_MOESM1_ESM.pdf]

Supplementary Information:

Supplementary Methods:

The figures shown below provide additional data to compliment the Methods section of the main text.

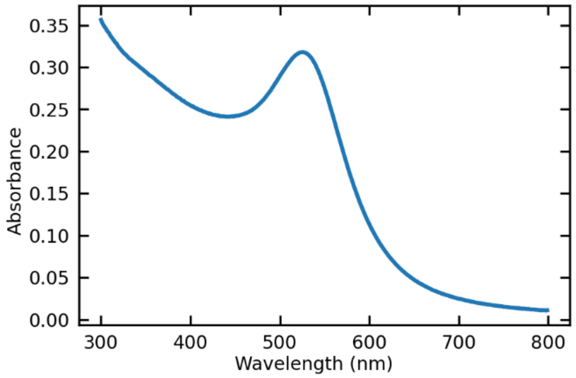

Supplementary Figure 1: UV-Vis spectra of a diluted aliquot of synthesized Brust stock Nanoparticles[1,2] described in the Methods section of the main text.

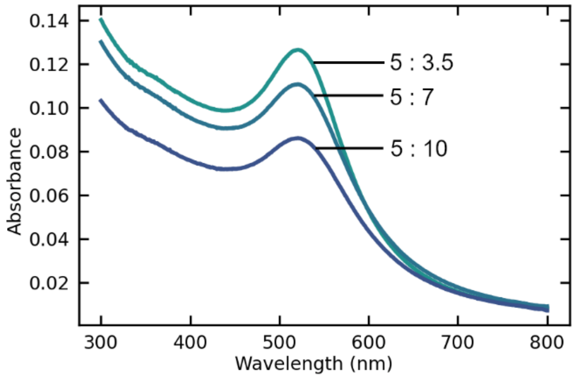

Supplementary Figure 2: UV-Vis spectra of size-selected NPs in hexanes[3] as described in the Methods section of the main text. Labels indicate the volume ratios of toluene : DDA/EtOH at which the NPs were fractionalized.

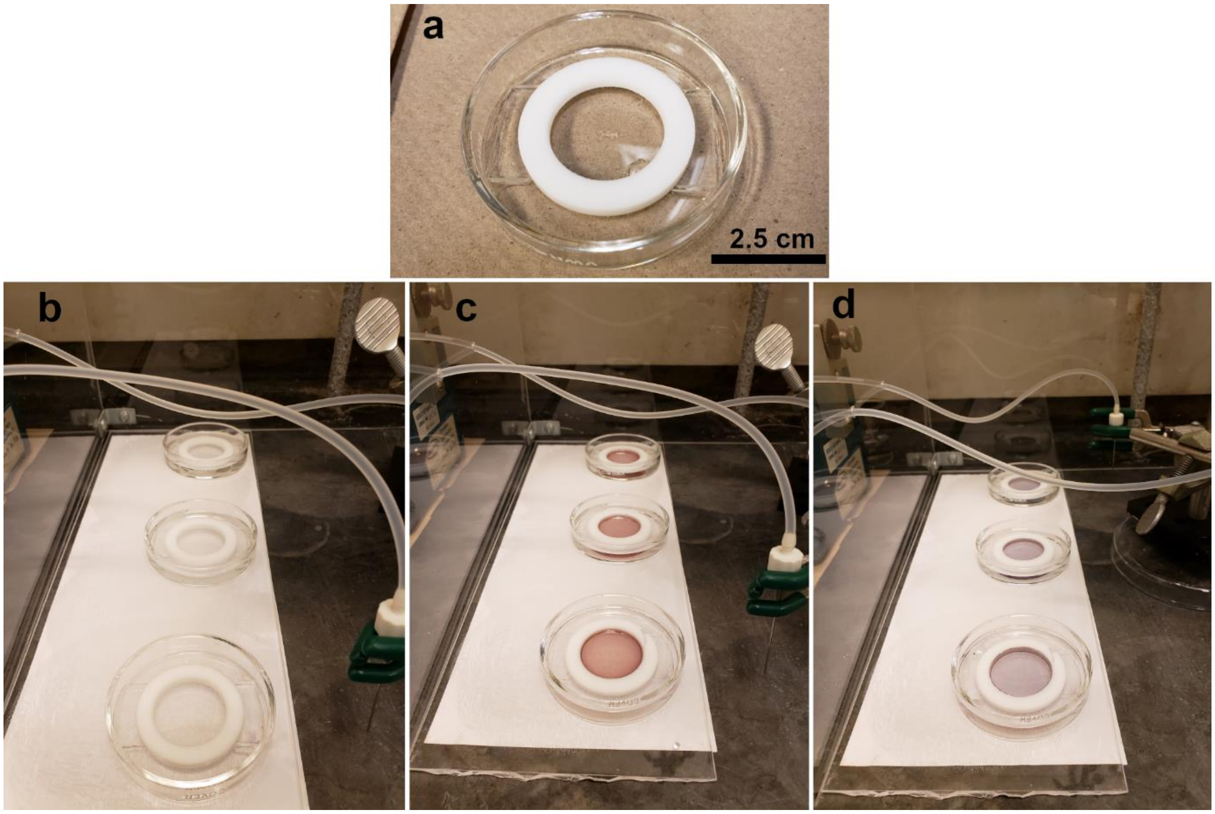

Supplementary Figure 3: Photographs of Teflon ring apparatus[3,4] and self-assembled NP films at the air-water interface as described in the Methods section of the main text. a) A single Teflon ring (inner diameter = 2.51 cm) supported on glass stands (cut microscope slides, x2 on each side of the ring) inside a Petri dish. b) Three Teflon ring apparatus as described in a), filled with water and prepared for NP solution drop casting. c) Evaporating NP solutions photographed within a minute of drop casting upon the water surfaces, contained within the three Teflon ring apparatus described in b). d) Self-assembled NP films generated from the solutions in c) 30 minutes after drop casting.

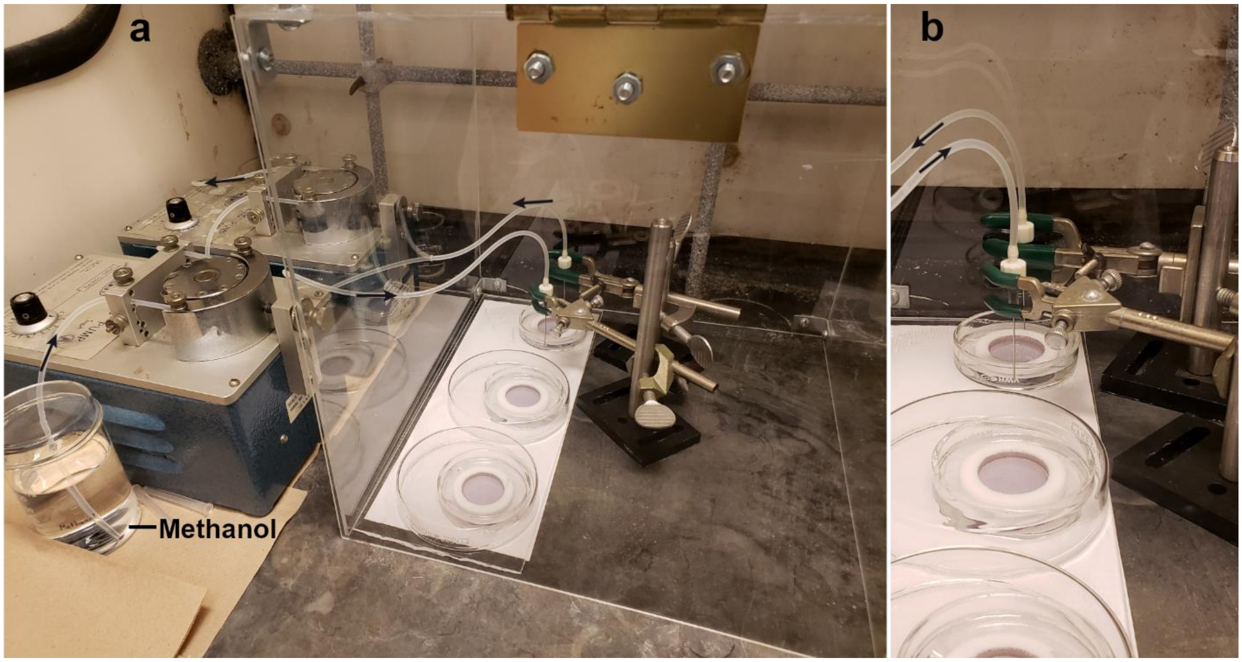

Supplementary Figure 4: Photographs of the pumping apparatus used to exchange water and methanol subphases supporting the NP films as described in the Methods section of the main text. Arrows depict the direction of fluid flow. a) A peristaltic pump is used to flow methanol from a reservoir into the system at ~1.2 mL/min, injecting beneath the liquid (water) surface which supports a self-assembled NP film. Simultaneously a secondary peristaltic pump is used to remove the methanol/water mixture beneath the NP film at the same flow rate. The NP films and Teflon ring apparatus are housed within a transparent Plexiglass box. b) A magnified image of a NP film shown in a), displaying the stands and clamps securing tubes and 22G gauge needles used for methanol/water flow.

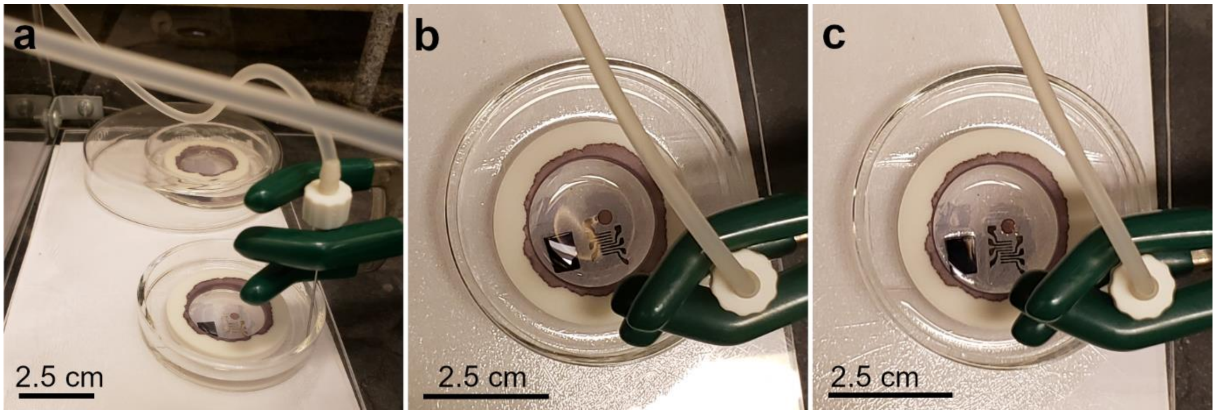

Supplementary Figure 5: Photographs of NP films during methanol subphase draining and deposition onto substrates as described in the Methods section of the main text (a TEM grid, patterned gold electrodes on a glass slide, and a quartz substrate are shown). a) A NP film cross-linked with octane-dithiol after ~1 minute of draining. b) A top-down view of the NP film shown in a). c) The NP film shown in b) after ~4 minutes of draining. The methanol subphase is pinned around the edges of the solid substrates, and the NP film has begun to contact the substrates.

Supplementary Data:

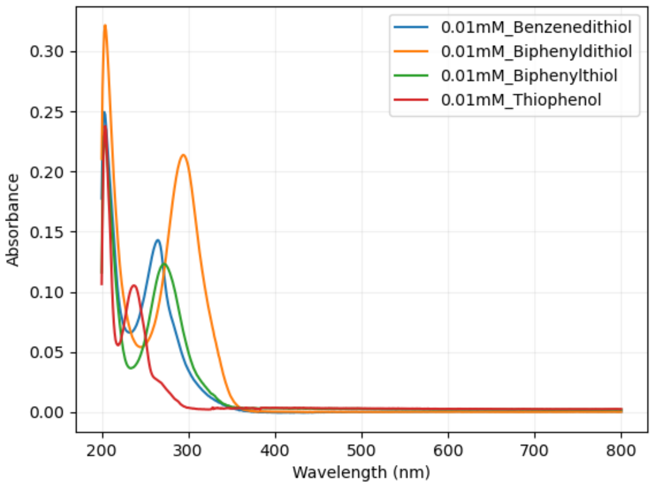

Supplementary Figure 6: UV-Vis absorption spectra for oligophenylene monothiols and dithiols in methanol.

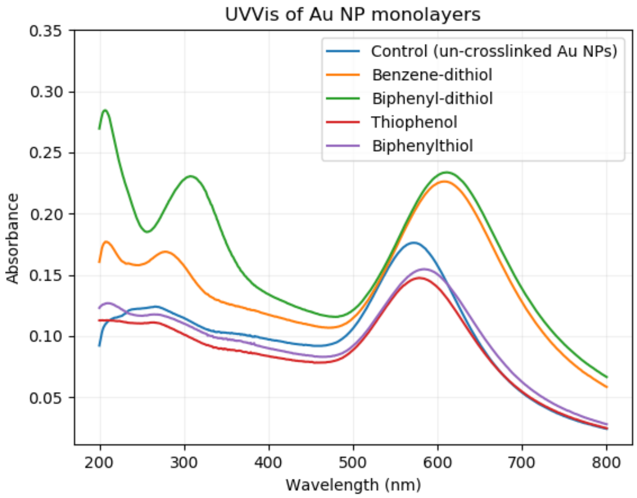

Supplementary Figure 7: UV-Vis spectra of NP films and X-NS collected on quartz substrates before normalization.

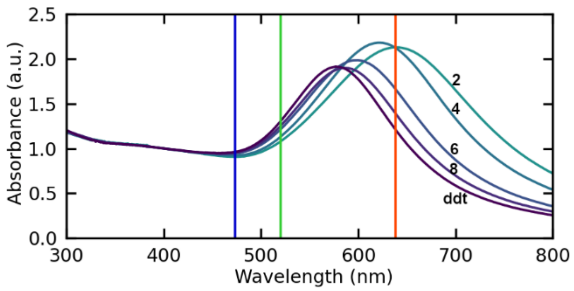

Supplementary Figure 8: Normalized UV-Vis absorbance spectra of NP films and X-NS on quartz prepared using dodecanethiol (ddt) and alkanedithiols (HS-$(CH_2)_n$-SH with n = 2, 4, 6, 8).

	Resistance (MΩ)					
**Film**	**Dodecane-thiol**	**Benzene-thiol**	**Biphenyl-thiol**	**Benzene-dithiol**	**Biphenyl-dithiol**	**Terphenyl-dithiol**
1	11300	2050	1230	583	127	17.7
2	18900	1770	996	927	127	15.6
3	16400	1200	751	619	113	12.1
4	----------	1260	1100	560	168	14.7
5	----------	1330	1260	515	200	18.0

Supplementary Table 1: NP film and X-NS room temperature DC resistances for various samples

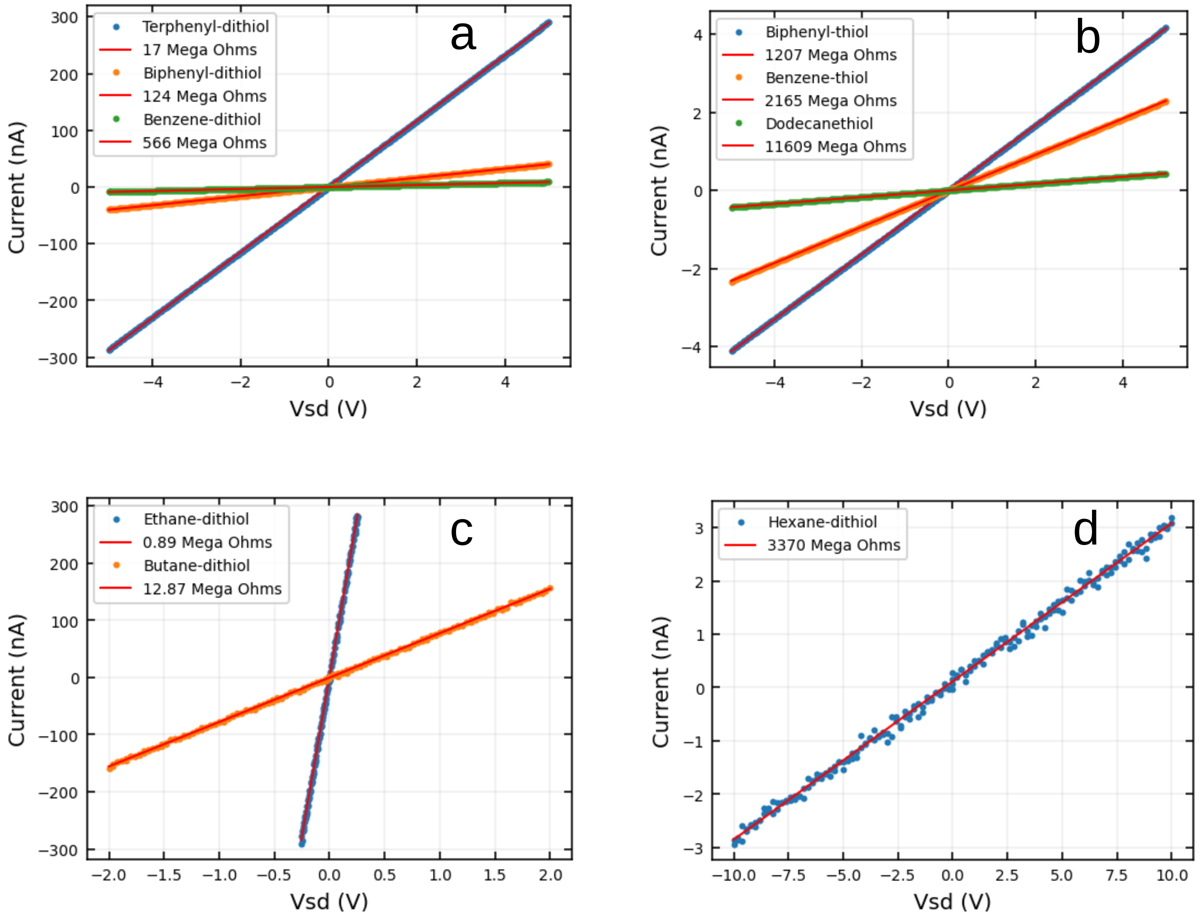

Supplementary Figure 9: Room temperature I-V curves of various Au NP monolayers and X-NS with linear fits fabricated with: a) terphenyl-dithiol, biphenyl-dithiol, and benzene-dithiol, b) biphenyl-thiol, benzene-thiol, and dodecanethiol, c) ethane-dithiol and butane-dithiol, and d) hexane-dithiol.

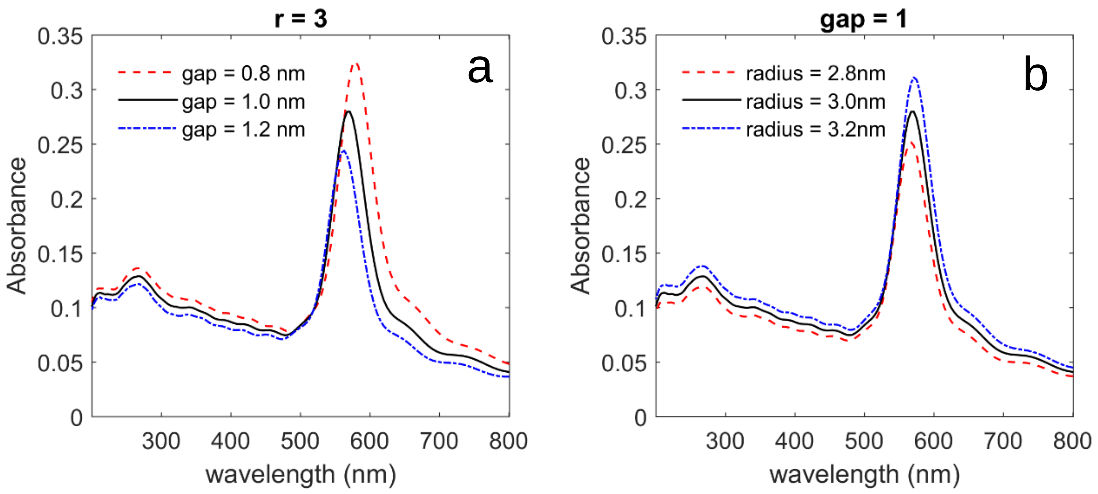

Supplementary Figure 10: Additional finite difference time domain modelling results. a) Calculated UV-Vis spectra for benzenethiol NP films showing the effect of varying NP-NP gap size for NPs of radius = 3 nm. b) Calculated spectra for varying NP sizes with a constant interparticle gap of 1 nm.

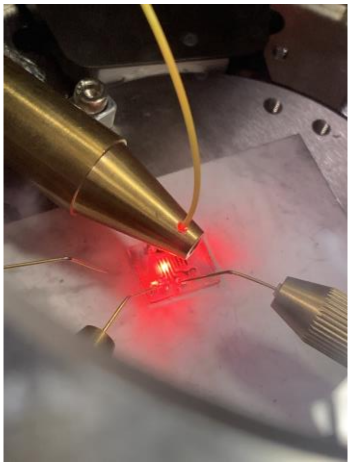

Supplementary Figure 11: Photograph showing the set up for photoconductivity measurements. Measurements are conducted at ~10^{-3} torr pressure and at room temperature. 2-probes are used, and a bias of 2V is applied during photoconductivity measurements.

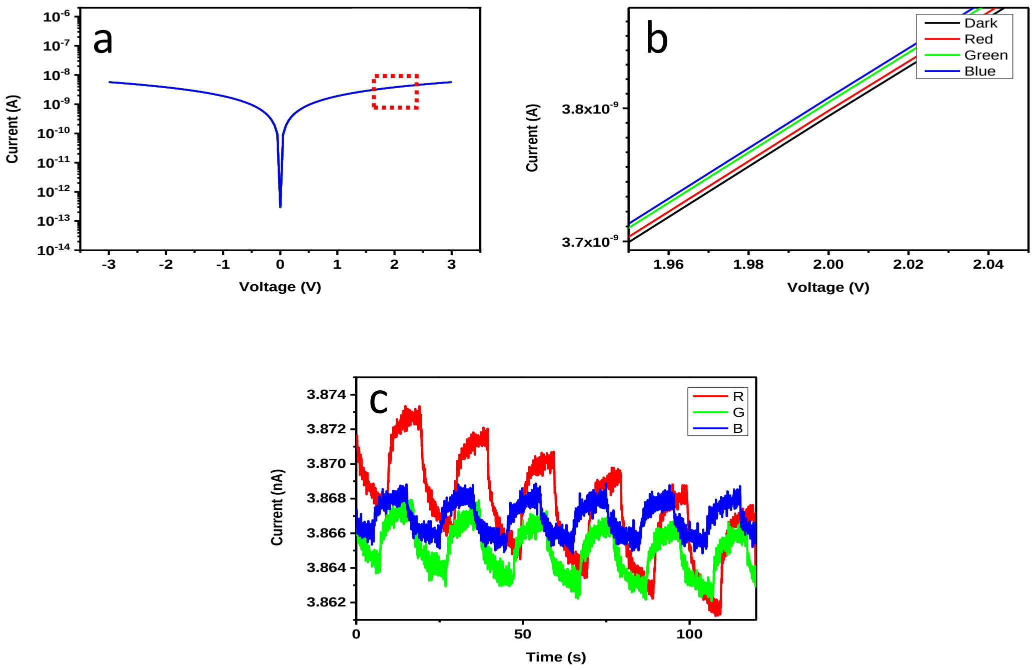

Supplementary Figure 12: Photocurrent measurements for benzene-dithiol X-NS. a) Current vs. bias voltage over $\pm$ 3V, b) current vs. bias voltage over a small range around 2V, and c) current at 2V vs. time measured as a laser illumination is repeatedly switched on/off. Excitation laser power/area and wavelengths are 78.2 mW cm^{-2} at 473 nm (blue data), 54.3 mW cm^{-2} at 520 nm (green data) and 28.2 mW cm^{-2} at 638 nm (red data). The spot diameter in all cases is ~3mm.

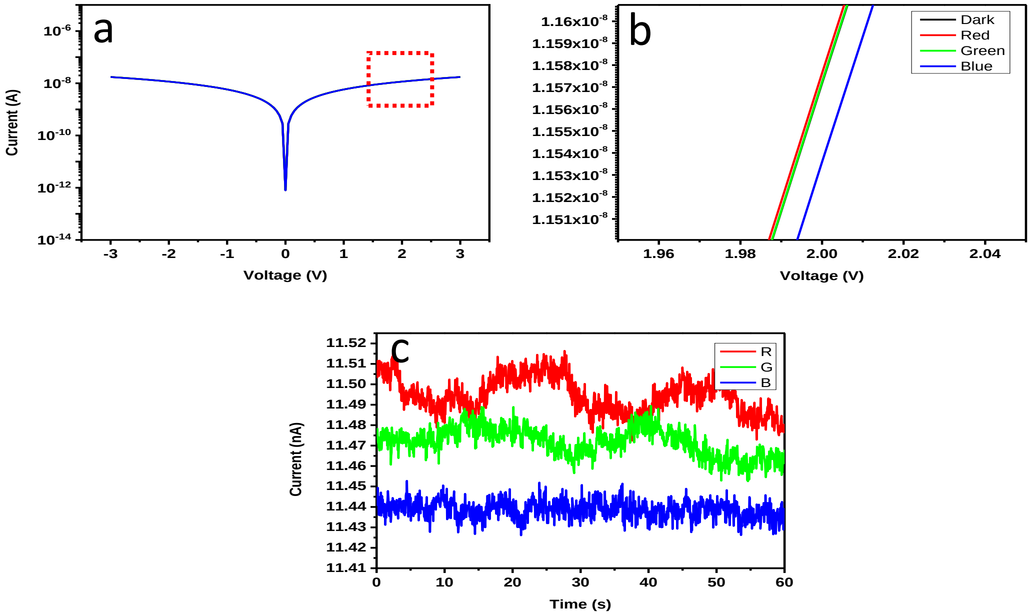

Supplementary Figure 13: Photocurrent measurements for biphenyl-dithiol X-NS. a) Current vs. bias voltage over ± 3V, b) current vs. bias voltage over a small range around 2V, and c) current at 2V vs. time measured as a laser illumination is repeatedly switched on/off. Excitation laser power/area and wavelengths are 78.2 mW cm^{-2} at 473 nm (blue data), 54.3 mW cm^{-2} at 520 nm (green data) and 28.2 mW cm^{-2} at 638 nm (red data). The spot diameter in all cases is ~3mm.

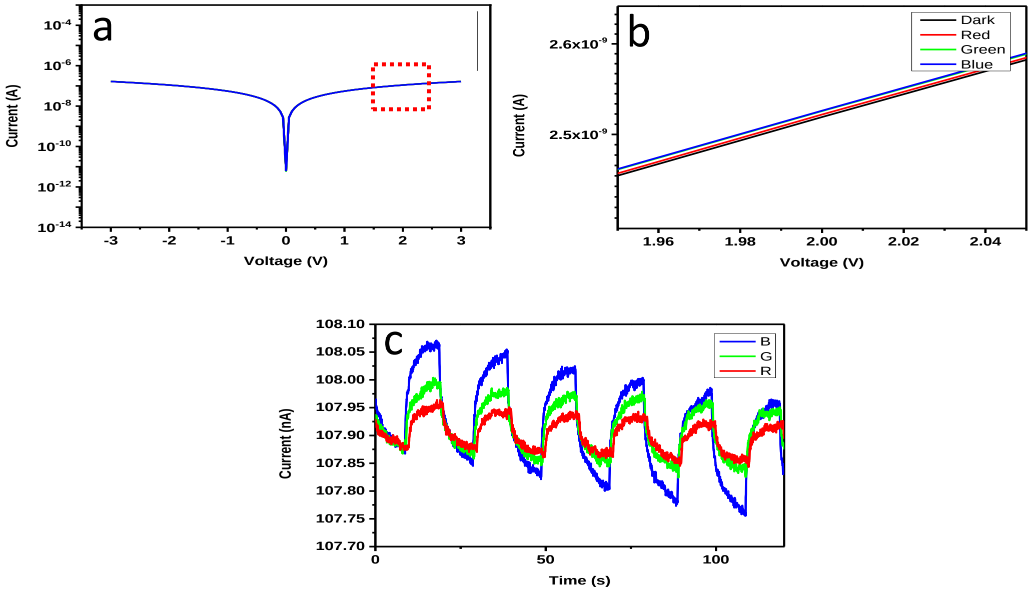

Supplementary Figure 14: Photocurrent measurements for terphenyl-dithiol X-NS. a) Current vs. bias voltage over ± 3V, b) current vs. bias voltage over a small range around 2V, and c) current at 2V vs. time measured as a laser illumination is repeatedly switched on/off. Excitation laser power/area and wavelengths are 78.2 mW cm^{-2} at 473 nm (blue data), 54.3 mW cm^{-2} at 520 nm (green data) and 28.2 mW cm^{-2} at 638 nm (red data). The spot diameter in all cases is ~3mm.

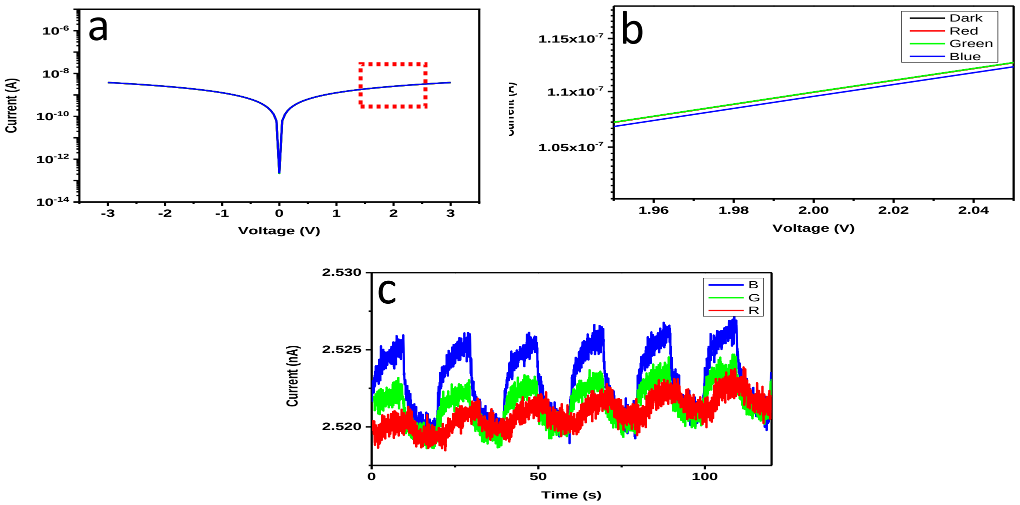

Supplementary Figure 15: Photocurrent measurements for a benzenethiol NP film. a) Current vs. bias voltage over ± 3V, b) current vs. bias voltage over a small range around 2V, and c) current at 2V vs. time measured as a laser illumination is repeatedly switched on/off. Excitation laser power/area and wavelengths are 78.2 mW cm^{-2} at 473 nm (blue data), 54.3 mW cm^{-2} at 520 nm (green data) and 28.2 mW cm^{-2} at 638 nm (red data). The spot diameter in all cases is ~3mm.

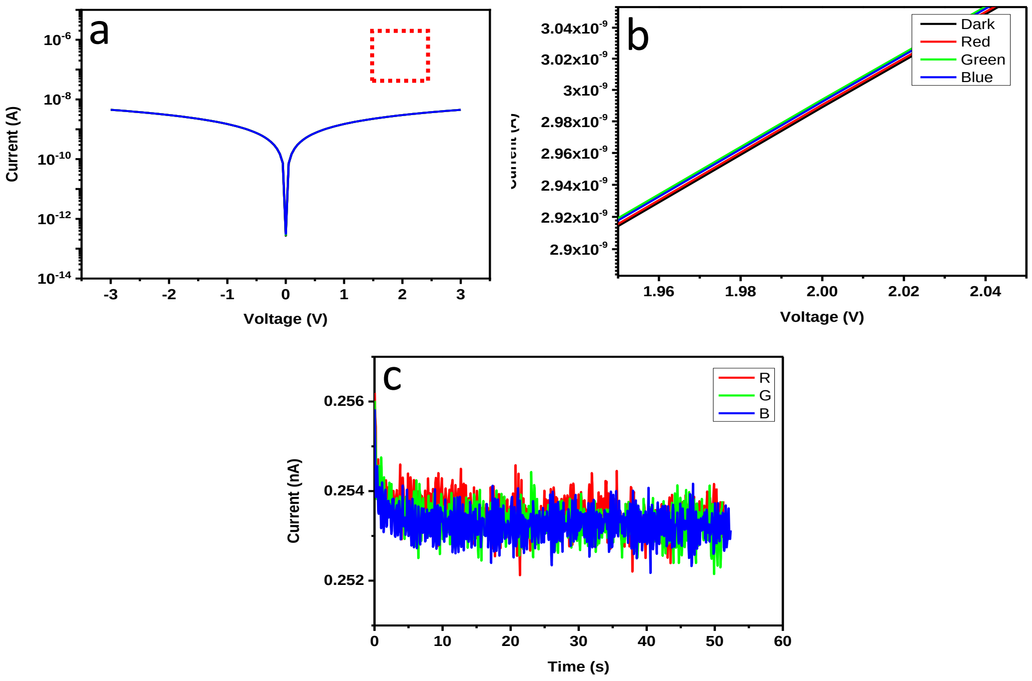

Supplementary Figure 16: Photocurrent measurements for a biphenylthiol NP film. a) Current vs. bias voltage over ± 3V, b) current vs. bias voltage over a small range around 2V, and c) current at 2V vs. time measured as a laser illumination is repeatedly switched on/off. Excitation laser power/area and wavelengths are 78.2 mW cm^{-2} at 473 nm (blue data), 54.3 mW cm^{-2} at 520 nm (green data) and 28.2 mW cm^{-2} at 638 nm (red data). The spot diameter in all cases is ~3mm.

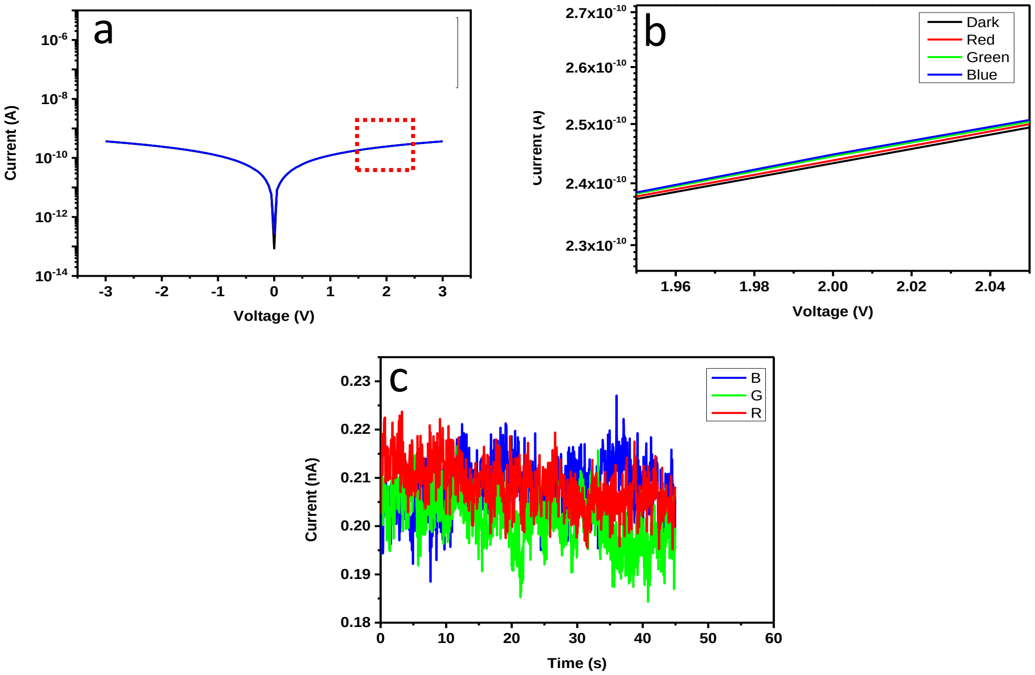

Supplementary Figure 17: Photocurrent measurements for a dodecanethiol NP film. a) Current vs. bias voltage over $\pm$ 3V, b) current vs. bias voltage over a small range around 2V, and c) current at 2V vs. time measured as a laser illumination is repeatedly switched on/off. Excitation laser power/area and wavelengths are 78.2 mW cm^{-2} at 473 nm (blue data), 54.3 mW cm^{-2} at 520 nm (green data) and 28.2 mW cm^{-2} at 638 nm (red data). The spot diameter in all cases is ~3mm.

Supplementary Notes:

1. Estimating the number of molecules in solution vs. X-NS film

Solution: From rearranging the Beer-Lambert law for a lossy medium, molecular absorbance is given by:

$$A = (\log_{10} e)\frac{4\pi\kappa_m l}{\lambda} = \varepsilon c l$$

where l is the path length of the light in the medium, c is the concentration of the molecule, ε is the molar absorptivity constant, λ is the wavelength of light and κ_m is the molecular extinction coefficient (complex component of the molecular complex index of refraction, N_m).

cl has units of moles/area, which gives a 2D footprint of molecule density which can be more easily compared to the # of molecules in a monolayer of gold nanoparticles (Au NPs) either capped or cross-linked with thiols/dithiols, respectively.

Using

$$c = 0.01\, mM$$
$$l = 1cm$$

we find

$$cl \sim 60.2\, molecules/nm^2 \text{ in solution.}$$

XSANS: From the diagram below of hexagonally close-packed lattice, one can calculate the number of Au NPs per area as:

$$\frac{\#NP}{Area} = \frac{2}{\sqrt{3}(s+d)^2}$$

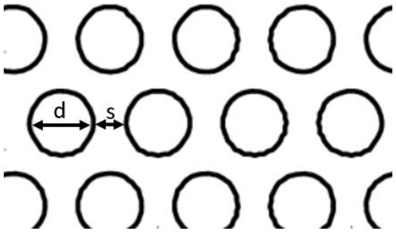

Using the packing densities, p, one can approximate the number of molecules per nanoparticle:

$$\frac{\#molecules}{NP} = p * 4\pi\left(\frac{d}{2}\right)^2 = \pi p d^2$$

Combining the above 2 equations gives the number of molecules per unit area in a Au NP monolayer:

$$\frac{\#molecules}{Area} = \frac{2\pi p d^2}{\sqrt{3}(s+d)^2}.$$

Using

$$s \approx 1nm$$

$$d \approx 6nm$$

$$p_1 = 4.27\ molecules/nm^2 \text{ on Au(111) for benzenthiol}^5$$

$$p_2 = 4.63\ molecules/nm^2 \text{ on Au(111) for biphenyl-thiol}^5$$

we find

$$\frac{\#molecules}{Area} \approx 12.3 \frac{molecules}{nm^2} \textit{ for biphenyl-thiol}$$

$$\approx 11.4 \frac{molecules}{nm^2} \textit{ for benzenethiol (thiophenol)}$$

In summary, there are 5.5 – 6 times more molecules/area in 0.01M solution than in Au NP films and X-NS.

Supplementary References:

1) Brust, M.; Walker, M.; Bethell, D.; Schiffrin, D. J.; Whyman, R. Synthesis of Thiol-Derivatised Gold Nanoparticles in a Two-Phase Liquid-Liquid System. *J. Chem. Soc., Chem. Commun.*, **1994**, 801.
2) Fishelson, N.; Shkrob, I., Lev, O.; Gun, J.; Modestov, A. D. Studies on Charge Transport in Self-Assembled Gold-Dithiol Films: Conductivity, Photoconductivity, and Photoelectrochemical Measurements. *Langmuir* **2001**, 17, 403.
3) Gravelsins, S.; Dhirani, A.-A. A Rapid, High Yield Size-Selective Precipitation Method for Generating Au Nanoparticles in Organic Solvents with Tunably Monodisperse Size Distributions and Replaceable Ligands. *RSC Adv.,* **2017**, 7, 55830.
4) Santhanam, V.; Liu, J.; Agarwal, R.; Andres, R. P.; Self-Assembly of Uniform Monolayer Arrays of Nanoparticles. *Langmuir* **2003**, 19, 7881.
5) Azzam, W. *Self-Assembled Monolayers on Gold Made from Organothiols Containing an Oligophenyl-Backbone*. Ph.D. Thesis, Ruhr-University Bochum, Bochum, Germany, **2003**.
